# A Single Bout of Remote Ischemic Preconditioning Suppresses Ischemia-Reperfusion Injury in Asian Obese Young Men

**DOI:** 10.3390/ijerph20053915

**Published:** 2023-02-22

**Authors:** Min-Hyeok Jang, Dae-Hwan Kim, Jean-Hee Han, Jahyun Kim, Jung-Hyun Kim

**Affiliations:** 1 Department of Physical Education, General Graduate School, Kyung Hee University, Yongin-si 17104, Republic of Korea; 2 Department of Kinesiology, California State University Bakersfield, Bakersfield, CA 93311, USA; 3 Department of Sports Medicine, Kyung Hee University, Yongin-si 17104, Republic of Korea

**Keywords:** remote ischemic preconditioning, cutaneous blood flow, sympathetic reactivity, heart rate variability, obesity

## Abstract

Remote ischemic preconditioning (RIPC) has been shown to minimize subsequent ischemia-reperfusion injury (IRI), whereas obesity has been suggested to attenuate the efficacy of RIPC in animal models. The primary objective of this study was to investigate the effect of a single bout of RIPC on the vascular and autonomic response after IRI in young obese men. A total of 16 healthy young men (8 obese and 8 normal weight) underwent two experimental trials: RIPC (three cycles of 5 min ischemia at 180 mmHg + 5 min reperfusion on the left thigh) and SHAM (the same RIPC cycles at resting diastolic pressure) following IRI (20 min ischemia at 180 mmHg + 20 min reperfusion on the right thigh). Heart rate variability (HRV), blood pressure (SBP/DBP), and cutaneous blood flow (CBF) were measured between baseline, post-RIPC/SHAM, and post-IRI. The results showed that RIPC significantly improved the LF/HF ratio (*p =* 0.027), SBP (*p =* 0.047), MAP (*p =* 0.049), CBF (*p =* 0.001), cutaneous vascular conductance (*p =* 0.003), vascular resistance (*p =* 0.001), and sympathetic reactivity (SBP: *p =* 0.039; MAP: *p =* 0.084) after IRI. However, obesity neither exaggerated the degree of IRI nor attenuated the conditioning effects on the measured outcomes. In conclusion, a single bout of RIPC is an effective means of suppressing subsequent IRI and obesity, at least in Asian young adult men, does not significantly attenuate the efficacy of RIPC.

## 1. Introduction

Remote ischemic preconditioning (RIPC), defined as a set of brief events of ischemia-reperfusion applied in distant tissues or organs from the heart, has been known to protect the cardiovascular systems from subsequent ischemic events in animal [1,2] and human [3] studies. Therefore, ischemic preconditioning has been recognized as one of the non-invasive interventions to prevent ischemic reperfusion injury (IRI) that inevitably occurs during the recovery process after ischemia [4]. Although the signaling mechanism of RIPC remains unclear, the protective effect of RIPC is known to be attributed to both humoral and neural pathways [5].

However, the effectiveness of an acute bout of RIPC is still controversial. For example, a single bout of RIPC was shown to attenuate myocardial ischemic stress through the modification of autonomic nervous system activity in an animal model [6]. In human studies, the RIPC intervention was reported to suppress sympathetic elevation and oxidative stress, together with an improved reactive hyperemic response after IRI in healthy humans [3,7] and attenuated myocardial tissue damage in patients with myocardial infarction [8]. However, others have reported that a single bout of RIPC alters neither autonomic function in young healthy individuals [9], nor cerebrovascular function in the elderly [10]. Further, the efficacy of RIPC was shown to be reduced in some clinical conditions such as type 2 diabetes mellitus [11]. Such inconsistent results, especially in humans, may be partly explained by different health profiles and/or cardiovascular disease risk factors among individuals [12].

Obesity is prevalent worldwide and a strong predictor of ischemic stroke [13] and myocardial infarction regardless of sex, age, and ethnicity [14]; it is also likely to be linked to ischemic diseases [15]. Considering these aspects, the protective effects of RIPC on obesity are worth investigating; however, only a few animal-based studies have been conducted, showing conflicting results. For example, RIPC was shown to offer protective effects on the ischemic livers of obese rats [16], whereas other studies found no meaningful RIPC-induced augmentation of myocardial functions in obese animal subjects [17,18]. Further, to the best of our knowledge, there is no previous study investigating the efficacy of RIPC in obese individuals; therefore, it is uncertain whether obesity influences RIPC outcomes in humans.

For the abovementioned reasons, the purpose of the present study was to investigate whether a single bout of RIPC preserves and/or mitigates vascular function and sympathetic reactivity after induced IRI and to compare the possible differences between obese and normal-weight individuals. We hypothesized that (1) a single bout of RIPC would reduce the degree of IRI and (2) obesity would reduce the degree of RIPC-induced preservation of vascular function after IRI.

## 2. Materials and Methods

### 2.1. Ethical Approval

The current study was conducted after the approval from the Institutional Review Board at Kyung-Hee University (KHGIRB-21-531) and conformed to the standard set by the Declaration of Helsinki. All participants provided written informed consent before their study participation.

### 2.2. Study Design

A total of sixteen male participants (8 normal weight, 8 obese) were recruited for the present study (Table 1). Because all participants in this study were Asian, obesity was defined based on Asia Pacific body mass index criteria equal to or greater than 25 kg/$m^{2}$ [19]. All participants completed a medical screening questionnaire and those who reported a presence or history of cardiovascular or metabolic disease were excluded from the study.

### 2.3. Experimental Procedure

**Figure 1:**
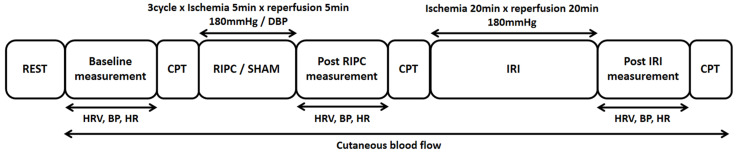
The schematic view of the experimental procedure.

The schematic view of the study procedure is presented in Figure 1. This study used a cross-over, repeated measures experimental design to test if a single bout of RIPC modifies the degree of IRI in the obese population. All participants visited the laboratory three times at one-week intervals after being abstained from alcohol and caffeine consumption and strenuous physical activity at least 24 h before the scheduled visits. During the first visit, the participants underwent health screening, demographic measurements, and experimental familiarization.

During the remaining two visits, RIPC and SHAM were performed in a counterbalanced order. For the experimental trials, the participants attired medical scrubs upon their arrival at the laboratory and remained in the supine position on a bed for instrumentation. A contoured inflation cuff (18 × 108 cm) was placed on the left proximal thigh for the implementation of RIPC and another cuff (24 × 122.5 cm) was placed on the right proximal thigh to induce IRI. Electrocardiogram standard limb leads (SE-1515, Edan Instruments Inc, Shenzhen, China) were placed onto the torso to monitor heart rate variability (HRV). Cutaneous blood flow was measured (perfusion unit: PU), using laser Doppler flowmetry (LDF, VMS-LDF2, Moor Instruments Ltd., Devon, England) throughout the experiment. The Doppler probe was attached 2 cm from the medial side of the great saphenous vein in the middle of the right leg tibia.

After the completion of the instrumentation, participants rested quietly in a supine position for 20 min followed by the experimental measurements, which were carried out three times throughout the test (e.g., Baseline, Post RIPC/SHAM, and Post IRI). The measurement started with cutaneous blood flow that was continuously monitored throughout the protocol but analyzed at a specific time point followed by HRV measurement for 5 min. Subsequently, blood pressure and pulse rate were measured using a digital sphygmomanometer (BP742N, OMRON Corporation, Kyoto, JAPAN) together with the cold pressor test (CPT) to determine sympathetic reactivity. CPT was carried out by the immersion of participants’ right hand in cold water (at 4–5 °C) for 3 min during which blood pressure and pulse rate were measured at the end of the first and third minutes.

Following the completion of the baseline measurement, RIPC or SHAM was carried out using a rapid cuff inflation system (E20, Hokanson Inc., Bellevue, WA, USA). The RIPC protocol consisted of 3 cycles of ischemia at 180 mmHg for 5 min and reperfusion for 5 min on the left leg. SHAM was performed in the same manner as RIPC; however, the compression intensity was set at each participant’s diastolic blood pressure measured at baseline. Post-RIPC measurement was started immediately after RIPC application followed by induction of the IRI on the right leg for 20 min of ischemia at 180 mmHg and 20 min of reperfusion. We judged that the ischemic stress threshold was reached when the cutaneous blood flow value fell below 20% compared with the baseline [20]. Finally, as soon as the reperfusion period was over, post-IRI measurements were taken in the order described above.

### 2.4. Calculation

Power spectral analysis of HRV (1600 Hz sampling frequency) was conducted using the fast Fourier transform and expressed as the ratio of the low frequency (0.04–0.15 Hz) to high frequency (0.15–0.4 Hz) (LF/HF ratio) to determine an overall balance between the sympathetic and parasympathetic activities. In addition, the lower limb cutaneous vascular conductance (CVC) and cutaneous vascular resistance (CVR) were calculated based on cutaneous blood flow and mean arterial pressure as shown below.

- CVC (PU/mmHg) = Cutaneous blood flow ÷ Mean arterial pressure × 100
- CVR (mmHg/PU) = Mean arterial pressure ÷ Cutaneous blood flow

### 2.5. Statistical Analyses

All data in this study were analyzed using SPSS (Ver. 26, IBM, Somers, NY, USA) and presented as mean and standard deviation. A two-way repeated measures ANOVA (2 conditions × 3 time points) with obesity as a between-subject factor was used to compare dependent variables between RIPC and SHAM. When a significant F-value was detected with Greenhouse–Geisser correction for sphericity, a post hoc pairwise comparison with Bonferroni correction was carried out to compare conditions at each time point. The significance level of all statistical analyses was set at α = 0.05.

## 3. Results

### 3.1. Heart Rate Variability, Blood Pressure, and Resting Heart Rate

**Figure 2:**
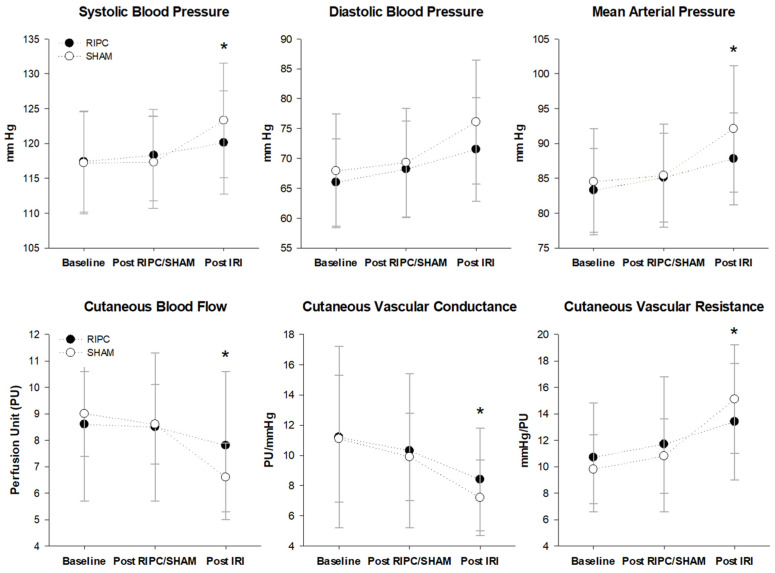
Hemodynamic response before and after induced ischemic-reperfusion injury between RIPC and SHAM (*n* = 16). Values are mean ± standard deviation. * Significant difference between groups (*p* < 0.05).

There was a significant interaction for the LF/HF after IRI in the RIPC compared with the SHAM (F = 4.631, *p* = 0.027) (Table 2). Consistently, SBP (F = 3.423, *p* = 0.047) and MAP (F = 3.488, *p* = 0.049) after IRI were significantly lower in RIPC compared with SHAM, but no difference was found for DBP (F = 1.698, *p* = 0.206) and HR (F = 0.589, *p* = 0.550) (Figure 2). However, the existence of obesity did not alter the effect of RIPC on these variables (LF/HF ratio: F = 0.050, *p =* 0.939; HR: F = 1.260, *p =* 0.301; SBP: F = 0.634, *p =* 0.537; MAP: F = 0.572, *p =* 0.564; DBP: F = 0.572, *p =* 0.564).

### 3.2. Sympathetic Reactivity

A significant interaction was found for sympathetic reactivity, where there was an attenuated SBP response to cold in RIPC compared with SHAM after IRI (F = 5.382, *p =* 0.039); however, no significant difference was found in MAP (F = 3.549, *p =* 0.084) or DBP (F = 1.546, *p =* 0.238). Similarly, the existence of obesity did not alter the sympathetic reactivity (SBP: F = 0.726, *p =* 0.411; MAP: F = 0.012, *p =* 0.913; DBP: F = 0.062, *p =* 0.808).

### 3.3. Cutaneous Vascular Responses

A significant interaction was found for CBF, where there was an alleviated reduction in CBF after IRI in the RIPC compared with SHAM (F = 10.111, *p =* 0.001). Consequently, a significantly increased CVC and decreased CVR were found after IRI in the RIPC (CVC: F = 7.828, *p =* 0.003; CVR: F = 10.576, *p =* 0.001). However, RHI did not differ between conditions (F = 0.716, *p =* 0.474) (Figure 2), and the existence of obesity did not influence any of the vascular variables (LDF: F = 0.231, *p =* 0.784; CVC: F = 0.185, *p =* 0.819; CVR: F = 0.032, *p =* 0.962; RHI: F = 0.973, *p =* 0.381).

## 4. Discussion

To our knowledge, this is the first study investigating the effectiveness of RIPC in obese humans. It was found that a single bout of RIPC significantly suppressed the IRI-induced aggravation of vascular function and sympathetic reactivity compared with SHAM. However, there was no difference in any outcome measures between obese and normal-weight individuals. These results suggest that a single bout of RIPC is an effective means of mitigating injuries resulting from a subsequent ischemic event and such modifications were neither blunted nor magnified by obesity, at least in young adult males, in the present study.

The present results showed the significant inhibitory effects of RIPC on the reduction in CBF; in agreement with previous findings, these effects mitigated the IRI-related impairment in CVC and CVR. Kraemer et al. (2011) reported that a single bout of RIPC significantly increased blood flow and tissue oxygen saturation during the reperfusion phase in healthy young men [21]. Moreover, Kharbanda et al. (2002) showed that blood flow in response to acetylcholine after IRI alone was decreased, whereas a single bout of RIPC suppressed this reduction in healthy humans [1]. These acute preconditioning effects on vascular function, such as reduced coronary resistance and increased cerebral blood flow, have also been reported in animal studies [22,23,24]. Moreover, significantly suppressed elevation in SBP and MAP in RIPC (Figure 2), which might be explained by the augmented cutaneous vasodilation, supports previous findings [25] and implies therapeutic potential for blood pressure management [26].

When considering IRI-induced aggravation in vascular function is attributed to a decline in nitric oxide bioavailability and sympathetic-overactivation-induced vasoconstriction [27,28], a single bout of RIPC in the present study is thought to alter such impairments either singly or in combination. Although previous studies demonstrated RIPC-induced vasodilation originates from both endothelial-dependent and -independent vasodilators [29,30], the ability to explain which of the two vasodilation mechanisms was responsible for improved vascular function in the present experiment is limited. On the other hand, the suppressed vascular impairment in RIPC was accompanied by a significant attenuation in the LF/HF ratio (Table 2) and sympathetic reactivity to a cold stimulation after IRI (Figure 2), similar to the previous results of RIPC-induced improvement in sympathovagal balance in healthy humans [31] and patients with angina pectoris [25].

**Figure 3:**
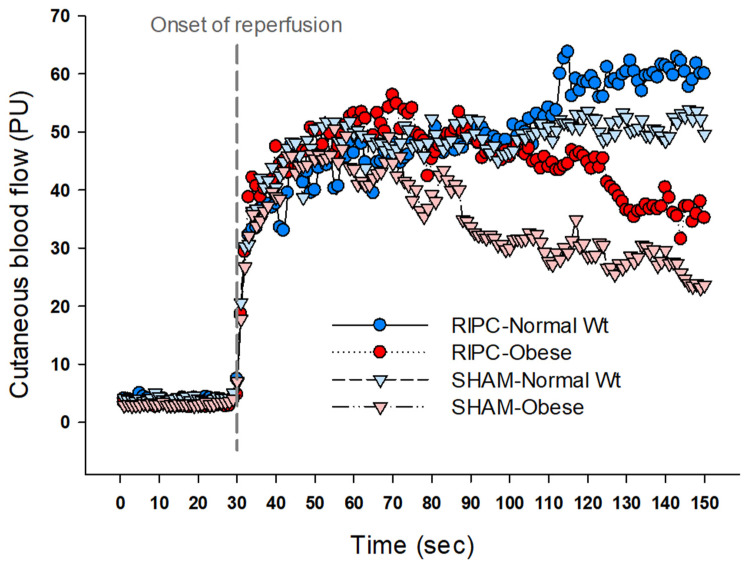
Reactive hyperemia responses after 20 min ischemia.

Both obesity and IRI are hypoxic and share similar inflammatory profiles including excessive production of reactive oxygen species and inflammatory cytokines [32,33]. Therefore, due to increased susceptibility to ischemic injury, we expected a greater degree of IRI together with reduced RIPC-induced preservation of vascular function in obese individuals compared with the normal-weight participants. Contrary to our expectations and previous results from the animal model [17,18], the present results showed the positive effects of RIPC on vascular and autonomic functions in obese participants after IRI, although the obese individuals showed a lesser degree of CBF recovery and maintenance over time compared with the normal-weight individuals in the first 2 min of reperfusion (Figure 3).

This might be due to the characteristics of the obese subjects participating in this study. Activation of phosphatase, known to limit the efficacy of both preconditioning and postconditioning with aging, was more pronounced in obese rats [34,35], but we recruited young healthy subjects without a history of cardiovascular and metabolic diseases. Regardless of the degree of BMI, the longer the period of obesity, the higher the risk of cardiovascular disease [36,37]. Their short obesity period and healthy physical condition most likely offset the adverse effects of obesity, such as cardiovascular disease and impaired physical function.

This study has several limitations. First, the present results and interpretation regarding obesity are limited to Asian men. Secondly, we also excluded female participants to rule out the effect of hormonal changes on measurements such as HRV. Finally, any blood parameters that may have been responsible for explaining the mechanisms and/or effects of RIPC on the outcomes were not included. Therefore, the potential roles of some important markers such as inflammatory cytokines and various vasodilators were limited in the present interpretation.

## 5. Conclusions

A single bout of RIPC was found to be an effective means of reducing impairment in vascular function and hyper-sympathetic nerve activity resulting from an acute ischemic event. Further, based on the present outcome measures, obesity neither significantly aggravated the degree of IRI nor abolished the favorable effects of RIPC in Asian obese young men. Future studies are warranted to investigate how repeated bouts of RIPC could further influence the functioning of the vascular systems in diverse obese populations.

## Figures and Tables

**Table 1:** Summary of participant characteristics.

Variable	Normal Weight (*n* = 8)	Obese (*n* = 8)
Age (years) *	25.8 ± 1.0	23.4 ± 2.0
Height (m)	1.75 ± 0.1	1.75 ± 0.1
Weight (kg) *	74.5 ± 4.9	86.1 ± 5.1
Body mass index (kg/m^2^) *	24.4 ± 0.8	28.0 ± 1.7
Body fat (%) *	15.1 ± 3.5	26.7 ± 2.6
Waist circumference (cm) *	80.0 ± 3.0	93.2 ± 4.5
Total cholesterol (mg/dL) *	182.4 ± 31.7	214.4 ± 44.8
Resting mean arterial pressure (mmHg) *	83.5 ± 4.5	92.2 ± 5.5

Values are mean ± standard deviation (*n* = 16). * Significant difference between groups (*p* < 0.01).

**Table 2:** Summary of heart rate and heart rate variability response to RIPC and SHAM (*n* = 16).

		RIPC			SHAM		
		Baseline	Post-RIPC	Post-IRI	Baseline	Post-RIPC	Post-IRI
HR (bpm)	Normal	68.3 ± 9.2	64.7 ± 8.6	65.3 ± 7.6	61.1 ± 8.3	59.8 ± 8.2	63.6 ± 8.0
	Obese	71.1 ± 9.4	65 ± 6.3	66.6 ± 12	71.0 ± 5.8	68.4 ± 13.4	65.0 ± 8.7
LF/HF ratio	Normal	0.9 ± 0.5	1.2 ± 0.4	1.2 ± 0.4 *	1.1 ± 0.6	1.4 ± 0.9	2.1 ± 1.3 *
	Obese	2.2 ± 0.8	2.1 ± 0.9	1.6 ± 0.7 *	2.1 ± 1.6	2.1 ± 1.1	2.5 ± 2.0 *

Values are mean ± standard deviation. * Significant difference between conditions (*p* < 0.05).

## Data Availability

Not applicable.
